# SARS-CoV-2 Surveillance System in Canada: Longitudinal Trend Analysis

**DOI:** 10.2196/25753

**Published:** 2021-05-10

**Authors:** Lori Post, Michael J Boctor, Tariq Z Issa, Charles B Moss, Robert Leo Murphy, Chad J Achenbach, Michael G Ison, Danielle Resnick, Lauren Singh, Janine White, Sarah B Welch, James F Oehmke

**Affiliations:** 1 Buehler Center for Health Policy and Economics Feinberg School of Medicine Northwestern University Chicago, IL United States; 2 Feinberg School of Medicine Northwestern University Chicago, IL United States; 3 Institute of Food and Agricultural Sciences University of Florida Gainsville, FL United States; 4 Institute of Global Health Feinberg School of Medicine Northwestern University Chicago, IL United States; 5 Divison of Infectious Disease Feinberg School of Medicine Northwestern University Chicago, IL United States; 6 International Food Policy Research Institute Washington, DC United States

**Keywords:** global COVID surveillance, COVID-19, COVID-21, new COVID strains, Canada Public Health Surveillance, Great COVID Shutdown, Canadian COVID-19, surveillance metrics, wave 2 Canada COVID-19, dynamic panel data, generalized method of the moments, Canadian econometrics, Canada SARS-CoV-2, Canadian COVID-19 surveillance system, Canadian COVID transmission speed, Canadian COVID transmission acceleration, COVID transmission deceleration, COVID transmission jerk, COVID 7-day lag, Alberta, British Columbia, Manitoba, New Brunswick, Newfoundland and Labrador, Northwest Territories, Nova Scotia, Nunavut, Ontario, Prince Edward Island, Quebec, Saskatchewan, Yukon

## Abstract

**Background:**

The COVID-19 global pandemic has disrupted structures and communities across the globe. Numerous regions of the world have had varying responses in their attempts to contain the spread of the virus. Factors such as public health policies, governance, and sociopolitical climate have led to differential levels of success at controlling the spread of SARS-CoV-2. Ultimately, a more advanced surveillance metric for COVID-19 transmission is necessary to help government systems and national leaders understand which responses have been effective and gauge where outbreaks occur.

**Objective:**

The goal of this study is to provide advanced COVID-19 surveillance metrics for Canada at the country, province, and territory level that account for shifts in the pandemic including speed, acceleration, jerk, and persistence. Enhanced surveillance identifies risks for explosive growth and regions that have controlled outbreaks successfully.

**Methods:**

Using a longitudinal trend analysis study design, we extracted 62 days of COVID-19 data from Canadian public health registries for 13 provinces and territories. We used an empirical difference equation to measure the daily number of cases in Canada as a function of the prior number of cases, the level of testing, and weekly shift variables based on a dynamic panel model that was estimated using the generalized method of moments approach by implementing the Arellano-Bond estimator in R.

**Results:**

We compare the week of February 7-13, 2021, with the week of February 14-20, 2021. Canada, as a whole, had a decrease in speed from 8.4 daily new cases per 100,000 population to 7.5 daily new cases per 100,000 population. The persistence of new cases during the week of February 14-20 reported 7.5 cases that are a result of COVID-19 transmissions 7 days earlier. The two most populous provinces of Ontario and Quebec both experienced decreases in speed from 7.9 and 11.5 daily new cases per 100,000 population for the week of February 7-13 to speeds of 6.9 and 9.3 for the week of February 14-20, respectively. Nunavut experienced a significant increase in speed during this time, from 3.3 daily new cases per 100,000 population to 10.9 daily new cases per 100,000 population.

**Conclusions:**

Canada excelled at COVID-19 control early on in the pandemic, especially during the first COVID-19 shutdown. The second wave at the end of 2020 resulted in a resurgence of the outbreak, which has since been controlled. Enhanced surveillance identifies outbreaks and where there is the potential for explosive growth, which informs proactive health policy.

## Introduction

### Background

On January 30, 2020, the World Health Organization officially declared the outbreak of SARS-CoV-2, the virus that causes COVID-19, a public health emergency of international concern [1]. The virus has disrupted communities across the globe [2]. Numerous factors, including public health policies, climate, population characteristics, governance, and sociopolitical factors, led to varying levels of success at controlling the spread of SARS-CoV-2 [3-7]. Despite sharing many similarities, COVID-19 could not have played out more differently between Canada and the United States [8]. Canada reported a total of 849,517 COVID-19 infections or 2235 cases per 100,000 population and 21,723 COVID-19 deaths or 57 deaths per 100,000 population as of February 22, 2021 [9,10]. In comparison, as of February 22, 2021, the United States had a total of 28,221,129 COVID-19 infections or 8598 per 100,000 population and 501,663 COVID-19 deaths or 153 deaths per 100,000 population [9,10]. The COVID-19 infection and death rates of the United States are 3.8 times and 2.7 times those of Canada’s, respectively. Understanding the context in which the COVID-19 pandemic occurs and the dynamics of the pandemic will inform not only Canada but also those nations struggling to control the multiple outbreaks, such as the United States. Unfortunately, existing surveillance suffers from reporting biases, undercounts, missing data, and data contamination.

### How the Pandemic Unfolded in Canada

**Figure 1:**
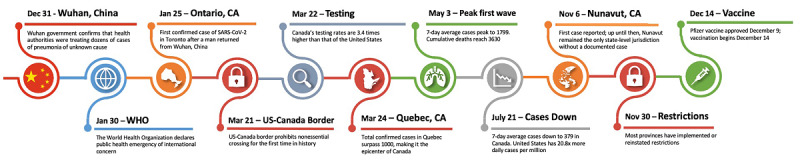
Timeline of major COVID-19 events for Canada. WHO: World Health Organization.

On January 25, 2020, Canada saw its first case of SARS-CoV-2 after a man returned to Toronto from Wuhan, China [11]. Cases in Ontario rose a month later [12]. Quebec soon became Canada’s first epicenter, likely due to southern travel into the United States during its winter school break, which occurred two weeks prior to lockdown measures implemented in mid-March [5]. Figure 1 shows the timeline of COVID-19 in Canada.

Fast forward one year later, Canada has had a resurgence of COVID-19 infections and has reimplemented public health guidelines to control the COVID-19 epidemic. As of February 22, 2021, Ontario and Quebec account for 68% of confirmed cases and 79% of deaths in Canada [10]. Nunavut, with a population of 38,780, remained the only geographical jurisdiction in North America without a single confirmed case of SARS-CoV-2 until early November 2020, when it began to see cases accelerate quickly [13]. Nunavut has one hospital and struggles with food security [14] and tuberculosis outbreaks [15]. Nunavut faced potentially catastrophic consequences from the introduction of SARS-CoV-2 to its region. Strict prevention measures barred anyone but residents and critical workers from entering—with required self-isolation for 14 days prior to entering [16]. The measures put in place by the Nunavut government were not entirely unique among its neighboring provinces and territories. The Northwest Territories had even stricter isolation measures, barring all travel to the territory except for residents, who were required to quarantine for 14 days in a government-run location [16]. Despite the substantial administrative authority each province possesses, Canadian leaders worked well together, constructing a unified response [17-19]. Across the country, mass gatherings were prohibited, schools and nonessential businesses were required to close, and fines were implemented for failing to follow social distancing policies [18,19]. Furthermore, there has been consistent messaging in regard to public health guidelines such as mask wearing [18]. This is in stark contrast to the United States, which has no national policies and failed to issue clear guidance on mask wearing, quarantines, hand hygiene, and social distancing, as many states have not implemented public health guidelines or supported the benefits of mask wearing [20-23].

### Politics

Furthermore, Canada’s response to the COVID-19 pandemic and social unity in public health adherence has likely benefited from the country's experience with the 2003 severe acute respiratory syndrome (SARS) epidemic that killed 44 Canadians [17]. A number of improvements were made in response to the SARS dress rehearsal in 2003, creating better federal-provincial collaboration and more effective public health communication strategies [17].

Canadian Prime Minister Justin Trudeau did not downplay the SARS-CoV-2 threat [18,24]. Trudeau began wearing a mask in May 2020 and practiced self-isolation for several weeks following his wife's diagnosis with SARS-CoV-2 in March [24,25]. Adherence to evidence-based guidelines that prevent the transmission of SARS-CoV-2 by political leaders is a critical component to promoting safe behavior and cooperation in the general population with public health guidelines [26]. Furthermore, in Canada, provincial leaders on polar ends of the political spectrum shared a cross-partisan consensus on how to manage the COVID-19 pandemic [18,19], whereas in the United States, political leaders of each political party have found little agreement on standard pandemic control measures including stay-at-home orders and mask mandates.

### Significance

Ideally, the development of a more advanced methodology for tracking and estimating COVID-19 transmission in regions within Canada will allow for more reliable analysis of which policies are effective and what other factors may be associated with transmission rates. Public health departments, as well as several universities and media outlets, are tracking the novel coronavirus using raw data including the number of new infections, testing, positivity, Rho, and deaths, in addition to other measures such as local hospital capacity [27-36]. To remove temporal effects, many surveillance systems have shifted to 7-day moving averages to counter the dearth of reporting during holidays and weekends. Although moving averages temper volatility of data and testing/reporting affects, surveillance still suffers from missing cases. General public health surveillance is helpful and provides a proxy of the pandemic, but surveillance data still suffer from significant bias due to undercounts, reporting delays, testing errors, dearth of testing, asymptomatic carriers, and other types of data contamination. In fact, surveillance systems are limited by the fact that they tend to include only the more severe cases while missing the mild cases and undiagnosed infections and deaths [37,38].

### Objective

To that end, the objective of our research is to provide standard surveillance metrics, which are necessary but not sufficient to detect the dynamics of the pandemic at the province level, even though they are limited to more severe cases and suffer from incomplete case ascertainment and data contamination. To address these data limitations, we validated additional novel surveillance metrics: (1) speed, (2) acceleration, (3) jerk (change in acceleration), (4) 7-day lag, and (5) 7-day persistence effect [39-43].

The basic question we are trying to inform is the following: how are we doing this week relative to previous weeks? From a public health perspective, in the midst of a pandemic, we would like (at least) three affirmative responses: (1) there are fewer new cases per day this week than last week, (2) the number of new cases is declining from day to day, and (3) the day-to-day decline in the number of cases is even bigger this week than last week. Additionally, we would like some indicative information about significant shifts in how the pandemic is progressing—positive shifts could be the first indicators of the emergence of a new or recurrent hotspot, and negative shifts could be the first indicators of successful public health policy.

We use a longitudinal trend analysis study design in concert with dynamic panel modeling and the method of moments to correct for existing surveillance data limitations [39,40]. Specifically, we will measure significant weekly shifts in the transmission of COVID-19 (ie, increase, decrease, or plateau). Our study will measure the underlying causal effect from a given prior week that persists through the following week, with a 7-day persistence rate to explain a clustering/declustering effect. The 7-day persistence represents an underlying disease transmission wave, where a large number of transmissions in a given prior week that results in a large number of infections the following week then “echoes” forward into a large number of new transmissions and hence a large number of new cases 7 days forward.

In summary, we will measure negative and positive shifts in the transmission of SARS-CoV-2 and the dynamics of the pandemic with acceleration/deceleration and jerk. Our novel indicator of persistence does not suffer from sampling bias. Our surveillance system measures the dynamics of the pandemic so that provincial governments can be proactive rather than reactive to outbreaks at the subnational level. This informs decision making regarding disease control, mitigation strategies, and reopening policies as Canada continues to manage this pandemic.

## Methods

The Government of Canada compiles data from each of the provinces and territories. Data have been accessed daily through their official government website [44] since January 2020 and static measures are updated. Persistence and shifts in the pandemic are calculated biweekly. The current panel included 13 provinces and territories to include the entire country of Canada, with 62 days in each panel (n=806). An empirical difference equation was specified in which the number of new positive cases in each province each day is a function of the prior number of cases, the level of testing, and weekly shift variables that measure whether the outbreak was growing faster than, slower than, or at the same rate as the previous weeks. This resulted in a dynamic panel model that was estimated using the generalized method of moments (GMM) approach by implementing the Arellano-Bond estimator in R [45].

Dynamic panel models allow us to derive novel metrics of COVID-19 transmissions by using data collected from the government. Hence, we can provide additional useful metrics that are less labor intensive. Although this is less of a benefit for Canada because of the notable health system and immense resources, dynamic panel models are useful in low-income countries that do not have the resources to have teams of epidemiologists model COVID-19. Dynamic panel data using GMM are the same methods used to measure the expansion and contraction of the economy. These methods lend themselves well to measuring the pandemic. Given the extensive detail necessary to derive novel metrics that are necessary for this study, we refer to original works by Oehmke and colleagues [40], followed by a proof of concept of a surveillance system with enhanced surveillance techniques to measure the dynamics of the pandemic [39].

Existing surveillance metrics are helpful at gauging the spread of COVID-19 and to that end, we provide traditional measures that include the number of new COVID-19 cases, cumulative cases since the outbreak began, a 7-day moving average to control for temporal effects such as dearth of reporting during weekends and holidays, the rate of infection per 100,000 population, new daily number of deaths, cumulative number of deaths, the 7-day moving average of deaths, and the death rate per 100,000 population. We consider these metrics as a proxy for the ongoing epidemic as surveillance systems tend to pick up on the more severe cases.

The dynamic measures include a temporal element to better understand how past cases affect the present and present cases affect the future. Dynamic measures include the following: (1) speed (the number of new observed COVID-19 cases per day per 100,000, averaged over a week), (2) acceleration (the change in speed from the prior week to the current week), (3) jerk (the week-over-week change in acceleration as a function of time over the course of 2 weeks), and (4) 7-day persistence effect on speed (the average of the number of new cases per day in a given week that are statistically attributable to new cases seven days earlier). We compare the week of February 7-13, 2021, with the week of February 14-20, 2021.

## Results

### Country Regression Results

Regression results for 13 Canadian provinces and territories are presented in Table 1. Weekly surveillance data in Tables 2-6 are based on these regressions. The regression Wald statistic is significant (*χ*^2^_8_= 615.617, *P*<.001). The Sargan test is not significant, failing to reject the validity of overidentifying restrictions, which further supports our model (*χ*^2^_511_=13, *P*=.99). The coefficient on the 7-day lag was both positive and statistically significant (*P*=.01), demonstrating that the number of infections one week prior to data collection had a significant effect on number of infections at the point of data collection. The shift parameter coefficient 14 days prior was positive but not significant (.116, *P*=.49), suggesting that exogenous shift events did not have an effect on cases. The cumulative number of tests administered was significant (coefficient .001, *P*=.02).

**Table 1:** Arellano-Bond dynamic panel data model of COVID-19 dynamics at the province level in Canada.

Variable	Coefficient	Chi-square (*df*)	*P* value
7-day lag	.332	N/A^a^	.01
Cumulative tests	.001	N/A	.02
7-day lag shift	.116	N/A	.49
Wald statistic for regression	N/A	615.617 (2, 8)	<.001
Sargan statistic for validity	N/A	13 (2, 511)	.99

^a^N/A: not applicable.

Tables 2-5 show static and novel dynamic surveillance measures for the weeks of February 7-13 and February 14-20, 2021. Additional weeks can be found in Tables S1-S10 in the Multimedia Appendix 1. Canada as a whole had a decrease in 7-day average COVID-19 cases from 2728 per 100,000 during the week of February 7-13 to 2502 per 100,000 during the week of February 14-20 (Tables 2 and 3). As of February 20, 2021, Canada had a relatively low caseload of 843,284 infections and 21,630 deaths. There are 37,742,154 people that reside in Canada and relative to the United States, their rate of infection is much lower.

Between the weeks of February 7-13 and February 14-20, 2021, we found indicators demonstrating an increase from the first to the second week of 19,947 cumulative cases (Tables 2 and 3).

**Table 2:** Static surveillance metrics for the week of February 7-13, 2021.

Province	New cases	Cumulative cases	7-day moving average	Infection rate	Deaths	Cumulative deaths	7-day moving average of death	Death rate
Alberta	305	128,532	303.43	6.90	15	1775	10	0.34
British Columbia	0	72,750	433.43	0	0	1288	6.00	0
Manitoba	99	30,687	75.57	7.18	0	866	3.43	0
New Brunswick	16	1398	8.71	2.05	0	22	0.29	0
Newfoundland and Labrador	26	686	38.71	4.98	0	4	0	0
Northwest Territories	0	43	1	0	0	0	0	0
Nova Scotia	2	1592	1.14	0.20	0	65	0	0
Nunavut	5	308	1.29	12.71	0	1	0	0
Ontario	1300	284,887	1167.00	8.82	19	6651	24	0.13
Prince Edward Island	0	114	0.29	0	0	0	0	0
Quebec	1049	275,880	986.14	12.23	28	10,201	28.86	0.33
Saskatchewan	244	26,389	168.57	20.70	4	354	2.57	0.34
Yukon	0	71	0.14	0	0	1	0	0
Canada	3046	823,337	2727.86	8.01	66	21,228	75.14	0.17

**Table 3:** Static surveillance metrics for the week of February 14-20, 2021.

Province	New cases	Cumulative cases	7-day moving average	Infection rate	Deaths	Cumulative deaths	7-day moving average of death	Death rate
Alberta	380	130,727	313.57	8.59	6	1818	6.14	0.14
British Columbia	0	75,835	440.71	0	0	1327	5.57	0
Manitoba	94	31,329	91.71	6.82	3	882	2.29	0.22
New Brunswick	3	1420	3.14	0.38	0	24	0.29	0
Newfoundland and Labrador	38	901	30.71	7.28	0	4	0	0
Northwest Territories	0	47	0.57	0	0	0	0	0
Nova Scotia	4	1608	2.29	0.41	0	65	0	0
Nunavut	6	338	4.29	15.25	0	1	0	0
Ontario	1228	291,999	1016	8.33	28	6848	28.14	0.19
Prince Edward Island	0	114	0	0	0	0	0	0
Quebec	769	281,456	796.57	8.97	14	10,292	13	0.16
Saskatchewan	193	27,438	149.86	16.37	3	368	2	0.25
Yukon	0	72	0.14	0	0	1	0	0
Canada	2715	843,284	2502.14	7.14	54	21,630	57.43	0.14

Canada’s speed of infection decreased from 8.38 new cases per 100,000 per week the week of February 7-13, 2021, to 7.5 new cases per 100,000 per week the week of February 14-20, 2021. Nunavut’s speed increased dramatically from 3.3 to 10.9 per 100,000; Quebec’s speed decreased from 11.5 to 9.3 per 100,000; Saskatchewan decreased from 14.3 to 12.7 per 100,000 over the two-week period from February 7-20. Saskatchewan’s persistent rate decreased from 8.6 to 6.4 between February 7 and February 20, 2021 (Table 6). Canada had deceleration in new cases and a negative jerk from February 7 to February 20 (Tables 4 and 5). Looking at provinces individually, the overall countrywide pattern holds, as all provinces had acceleration of <1 or deceleration and jerk of <1 or negative jerk from February 7-20.

**Table 4:** Novel surveillance metrics for the week of February 7-13, 2021.

Province	Speed: daily positives per 100,000 (weekly average of new daily cases per 100,000)	Acceleration: day-to-day change in the number of positives per day, weekly average, per 100,000	Jerk: week-over-week change in acceleration, per 100,000	7-day persistence effect on speed (number of new cases per day per 100,000 attributed to new cases 7 days ago)
Alberta	6.86	–0.14	0.13	3.86
British Columbia	8.42	0	0.07	3.65
Manitoba	5.48	0.20	0.50	3.30
New Brunswick	1.12	0.07	0.11	0.88
Newfoundland and Labrador	7.42	0.63	–0.71	0.09
Northwest Territories	2.21	0	0	0.14
Nova Scotia	0.12	0.03	0.03	0.03
Nunavut	3.27	0.73	0.73	2.44
Ontario	7.92	–0.09	0.49	4.49
Prince Edward Island	0.18	0	0	0.04
Quebec	11.50	–0.26	–0.06	5.68
Saskatchewan	14.30	–0.23	0.72	8.58
Yukon	0.34	0	–0.34	0
Canada	8.38	–0.10	0.23	4.37

**Table 5:** Novel surveillance metrics for the week of February 14-20, 2021.

Province	Speed: daily positives per 100,000 (weekly average of new daily cases per 100,000)	Acceleration: day-to-day change in the number of positives per day, weekly average, per 100,000	Jerk: week-over-week change in acceleration, per 100,000	7-day persistence effect on speed (number of new cases per day per 100,000 attributed to new cases 7 days ago)
Alberta	7.09	0.24	0.21	3.07
British Columbia	8.56	0	–0.17	3.77
Manitoba	6.65	–0.05	–0.15	2.45
New Brunswick	0.40	–0.24	–0.26	0.50
Newfoundland and Labrador	5.88	0.33	0.05	3.32
Northwest Territories	1.27	0	0	0.99
Nova Scotia	0.23	0.03	0	0.05
Nunavut	10.89	0.36	–0.36	1.46
Ontario	6.90	–0.07	–0.14	3.54
Prince Edward Island	0	0	0	0.08
Quebec	9.29	–0.47	–0.16	5.15
Saskatchewan	12.71	–0.62	–0.11	6.40
Yukon	0.34	0	0.34	0.15
Canada	7.50	–0.12	–0.10	3.75

In general, between February 7, 2021, and February 20, 2021, the speed of the COVID-19 pandemic slowed slightly in Canada as it went from 8.4 to 7.5 cases per day per 100,000 population. The pandemic was decelerating during the week of February 13, 2021, by –0.1 cases per day per 100,000 population and maintained that minor deceleration the following week at –0.1 per day per 100,000 population. Moreover, comparing the acceleration week over week, the rate of acceleration jerked downward by –0.1 per day per 100,000 population.

**Table 6:** Provinces with the highest 7-day persistence.

Date and provinces		7-day persistence values
**February 13, 2021**		
	Saskatchewan	8.58
	Quebec	5.68
	Ontario	4.49
	Alberta	3.86
	British Columbia	3.65
**February 20, 2021**		
	Saskatchewan	6.40
	Quebec	5.15
	British Columbia	3.77
	Ontario	3.54
	Newfoundland and Labrador	3.32

The persistence rate, which is the number of cases this week that are statistically linked to novel infections a week ago, shows the pandemic shifting downward with the exception of British Columbia, which had a modest increase (Table 6). Less densely populated areas of Newfoundland and Labrador, the Northwest Territories, Nova Scotia, and Prince Edward Island experienced modest increases in the persistence of infections during the week of February 20, 2021.

The most populous provinces and their populations are shown in Table 7. Of the most populous provinces, Ontario and Quebec both had a decrease in speed, deceleration, and negative jerk during February 7-20, 2021. All dynamic indicators of the two largest provinces show that the pandemic is slowing. Of concern are British Columbia, Alberta, and Manitoba because they are headed in the opposite direction. Specifically, the speed of the pandemic is increasing in these three provinces. Acceleration of the daily speed, which is the week-to-week change in the number of new daily cases per 100,000 population increased for Alberta, remained at 0 for British Columbia, and decreased slightly for Manitoba the week of February 20, 2021. Although the speed is increasing, the day-to-day increase in COVID-19 transmissions jerked downward for British Columbia and Manitoba, with Alberta’s jerk slightly increasing (Tables 4 and 5). Jerk can be interpreted as a given week’s acceleration minus the prior week’s acceleration. Jerk helps us to understand how the pandemic is increasing or decreasing relative to last week. Nunavut’s rates indicated an outbreak between the weeks of February 7-13 and February 14-20, 2021, when the rate of speed increased from 3.3 to 10.9 new infections per day per 100,000 population. Fortunately, there is evidence that this outbreak is slowing. The acceleration of speed slowed during this time, down from 0.7 per 100,000 per day during the week of February 7-13 to 0.4 per 100,000 per day during the week of February 14-20. The persistence rate also decreased from 2.4 to 1.5 cases that are directly attributable to the number of new infections 7 days earlier. Although Nunavut experienced an outbreak, we also see the pandemic slowing, indicating a favorable response to mitigation efforts.

**Table 7:** Most populous Canadian provinces.

Province	Population as of 2020
Ontario	14,734,014
Quebec	8,574,571
British Columbia	5,147,712
Alberta	4,421,876
Manitoba	1,379,263

### Province Regression Results

**Figure 2:**
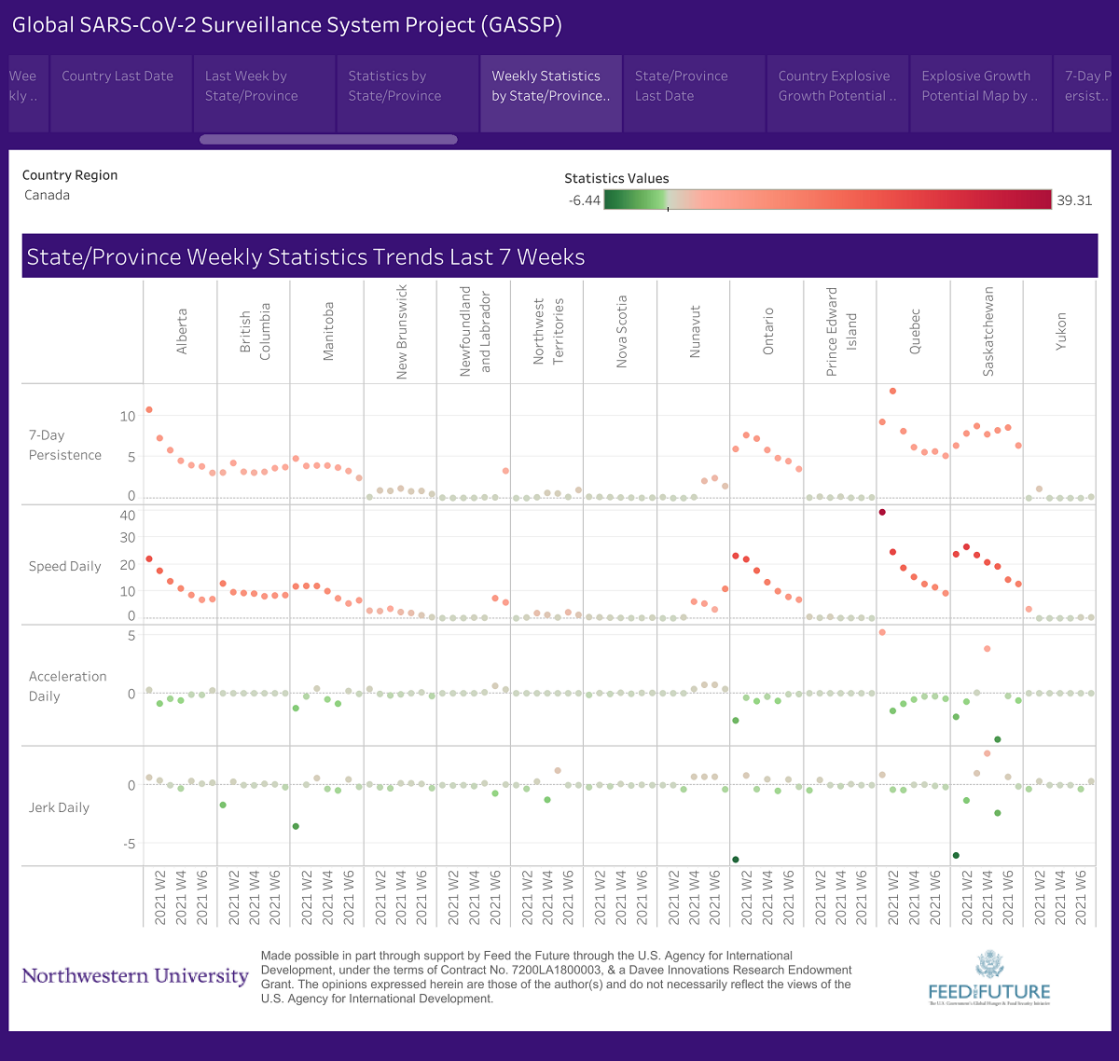
Screenshot of weekly Canada SARS-CoV-2 trends by province/territory.

Due to the nature of publications, the moment we publish our research findings, the data become outdated. To that end, the active surveillance system that informed this study, which contains more recent data, can be accessed online [46]. Figure 2 provides a visualization of our novel metrics over a 3-week period.

## Discussion

### Principal Findings

The novel metrics applied in this paper advance Canada’s surveillance capacity for monitoring the COVID-19 pandemic. In particular, acceleration and jerk provide a more complete picture of day-to-day and week-to-week changes in the pandemic than do traditional surveillance measures. The 7-day persistence rate helps identify areas where a high incidence of cases one week is passed forward to create a high incidence of cases the next week, and high persistence rates indicate an underlying problem with pandemic control. These measures, available through an online data dashboard, provide valuable information to Canada (as well as other countries) to monitor the pandemic.

Canada initially had an outbreak of COVID-19 around the same time as the initial outbreak in the United States. At many levels, the Unites States and Canada are similar in that they are both located in North America and both are developed, high-income countries, with vast geographical areas and heterogeneous populations. Beyond this, the similarities end between the Unites States and Canada as it relates to COVID-19 transmissions.

Canada developed a coordinated national plan between political parties and among its provinces and territories. First and foremost, Canada successfully implemented a widespread shutdown*,* which led to the initial reduction in COVID-19 transmissions. In addition, social isolation, distancing, masking, testing, and contact tracing have resulted in a coordinated effort that has mitigated the pandemic. Fortunately, this has worked well for a number of provinces and territories. Though Canada overall still has new daily cases, the pandemic is slowing based on measures of acceleration, jerk, and 7-day persistence rate, meaning cases and transmission were trending downward during the week of February 14-20. These rates were trending upward the week before. The most alarming metric is that speed in Nunavut increased from 3.3 new cases per day per 100,000 population to 10.9 cases per day per 100,000 population, which is a three-fold increase. This is indicative of an outbreak; however, the Canadian response to novel infections in Nunavut resulted in the speed decelerating, which is evident in the decrease in the acceleration rate as well as a negative jerk during the week of February 14-20, 2021.

Canada is successfully tamping down the pandemic, evident by the decrease in daily cases of daily transmissions from 3046 to 2715, concurrent with a decrease in the 7-day moving average, decrease in deaths, and decrease in death rates. Regardless, the weekly number of new cases remains at 2715 for the week of February 20, 2021. Such a large caseload could quickly escalate to a large outbreak if prevention efforts wane.

### Conclusion

Canada has maintained good COVID-19 control policies that resulted in fewer transmissions for the week of February 14-20, 2021, compared to the previous week; however, it is not time for Canada to declare victory over COVID-19 transmissions or to be complacent. The opposite is necessary. Canada must remain vigilant and continue implementing those policies that caused the Canadian outbreak to reverse course and decrease. In summary, we understand what causes COVID-19 and how it is transmitted. We also understand how to control outbreaks. Enhanced surveillance is the first indicator that an outbreak is occurring and that immediate action is needed. Conversely, enhanced surveillance also informs policy makers when policies put into place to control the COVID-19 pandemic are successfully decreasing the spread of disease.

## Appendix

Multimedia Appendix 1 Static and novel surveillance metrics for weeks between 1/3/2021-2/6/2021.

## Abbreviations

GMM: generalized method of moments
SARS: severe acute respiratory syndrome
