# Supplementary material for: SARS-CoV-2 Surveillance System in Canada: Longitudinal Trend Analysis
Source: JMIR Public Health Surveill. 2021 May 10;7(5):e25753. doi: 10.2196/25753 (PMC8112542; doi:10.2196/25753)
Supplement: Multimedia Appendix 1 [file publichealth_v7i5e25753_app1.docx]

**Table S1. Static surveillance metrics for the week of 1/3/2021-1/9/2021**

| Province | New cases | Cumulative cases | 7-day moving average | Infection rate | Deaths | Cumulative deaths | 7-day moving average of death | Death rate |
| --- | --- | --- | --- | --- | --- | --- | --- | --- |
| Alberta | 989 | 110633 | 973.29 | 22.37 | 31 | 1272 | 32.29 | 0.70 |
| British Columbia | 0 | 56632 | 663.14 | 0 | 0 | 988 | 12.43 | 0 |
| Manitoba | 203 | 26166 | 162.86 | 14.72 | 7 | 733 | 7.86 | 0.51 |
| New Brunswick | 30 | 765 | 22 | 3.84 | 0 | 9 | 0 | 0 |
| Newfoundland and Labrador | 0 | 392 | 0.29 | 0 | 0 | 4 | 0 | 0 |
| Northwest Territories | 0 | 24 | 0 | 0 | 0 | 0 | 0 | 0 |
| Nova Scotia | 3 | 1529 | 4.29 | 0.31 | 0 | 65 | 0 | 0 |
| Nunavut | 0 | 266 | 0 | 0 | 0 | 1 | 0 | 0 |
| Ontario | 3443 | 211837 | 3405.57 | 23.37 | 40 | 4922 | 42.43 | 0.27 |
| Prince Edward Island | 0 | 102 | 0.86 | 0 | 0 | 0 | 0 | 0 |
| Quebec | 3127 | 226233 | 3370.29 | 36.47 | 41 | 8647 | 60.14 | 0.48 |
| Saskatchewan | 329 | 17803 | 279.71 | 27.91 | 11 | 191 | 4.71 | 0.93 |
| Yukon | 0 | 70 | 1.43 | 0 | 0 | 1 | 0 | 0 |
| **Canada** | 8124 | 652452 | 7259.14 | 21.38 | 130 | 16833 | 159.86 | 0.34 |

**Table S2. Static surveillance metrics for the week of 1/10/2021-1/16/2021**

| Province | New cases | Cumulative cases | 7-day moving average | Infection rate | Deaths | Cumulative deaths | 7-day moving average of death | Death rate |
| --- | --- | --- | --- | --- | --- | --- | --- | --- |
| Alberta | 717 | 116079 | 778 | 16.21 | 15 | 1417 | 20.71 | 0.34 |
| British Columbia | 0 | 60117 | 497.86 | 0 | 0 | 1047 | 8.43 | 0 |
| Manitoba | 177 | 27322 | 165.14 | 12.83 | 1 | 761 | 4 | 0.07 |
| New Brunswick | 27 | 911 | 20.86 | 3.46 | 0 | 12 | 0.43 | 0 |
| Newfoundland and Labrador | 0 | 395 | 0.43 | 0 | 0 | 4 | 0 | 0 |
| Northwest Territories | 0 | 25 | 0.14 | 0 | 0 | 0 | 0 | 0 |
| Nova Scotia | 4 | 1554 | 3.57 | 0.41 | 0 | 65 | 0 | 0 |
| Nunavut | 0 | 266 | 0 | 0 | 0 | 1 | 0 | 0 |
| Ontario | 3056 | 234364 | 3218.14 | 20.74 | 51 | 5340 | 59.71 | 0.35 |
| Prince Edward Island | 0 | 104 | 0.29 | 0 | 0 | 0 | 0 | 0 |
| Quebec | 2225 | 240970 | 2105.29 | 25.95 | 67 | 9005 | 51.14 | 0.78 |
| Saskatchewan | 270 | 19985 | 311.71 | 22.91 | 2 | 212 | 3 | 0.17 |
| Yukon | 0 | 70 | 0 | 0 | 0 | 1 | 0 | 0 |
| **Canada** | 6476 | 702162 | 6144.57 | 17.04 | 136 | 17865 | 147.43 | 0.36 |

**Table S3. Static surveillance metrics for the week of 1/17/2021-1/23/2021**

| Province | New cases | Cumulative cases | 7-day moving average | Infection rate | Deaths | Cumulative deaths | 7-day moving average of death | Death rate |
| --- | --- | --- | --- | --- | --- | --- | --- | --- |
| Alberta | 573 | 120322 | 606.14 | 12.96 | 13 | 1525 | 15.43 | 0.29 |
| British Columbia | 0 | 63484 | 481 | 0 | 0 | 1128 | 11.57 | 0 |
| Manitoba | 216 | 28476 | 164.86 | 15.66 | 2 | 797 | 5.14 | 0.15 |
| New Brunswick | 17 | 1104 | 27.57 | 2.18 | 0 | 13 | 0.14 | 0 |
| Newfoundland and Labrador | 0 | 398 | 0.43 | 0 | 0 | 4 | 0 | 0 |
| Northwest Territories | 0 | 31 | 0.86 | 0 | 0 | 0 | 0 | 0 |
| Nova Scotia | 0 | 1570 | 2.29 | 0 | 0 | 65 | 0 | 0 |
| Nunavut | 0 | 267 | 0.14 | 0 | 0 | 1 | 0 | 0 |
| Ontario | 2359 | 252585 | 2603 | 16.01 | 52 | 5753 | 59 | 0.35 |
| Prince Edward Island | 0 | 110 | 0.86 | 0 | 0 | 0 | 0 | 0 |
| Quebec | 1685 | 252176 | 1600.86 | 19.65 | 76 | 9437 | 61.71 | 0.89 |
| Saskatchewan | 274 | 21917 | 276 | 23.25 | 3 | 250 | 5.43 | 0.25 |
| Yukon | 0 | 70 | 0 | 0 | 0 | 1 | 0 | 0 |
| **Canada** | 5124 | 742510 | 4845.14 | 13.48 | 146 | 18974 | 158.43 | 0.38 |

**Table S4. Static surveillance metrics for the week of 1/24/2021-1/30/2021**

| Province | New cases | Cumulative cases | 7-day moving average | Infection rate | Deaths | Cumulative deaths | 7-day moving average of death | Death rate |
| --- | --- | --- | --- | --- | --- | --- | --- | --- |
| Alberta | 383 | 123739 | 488.14 | 8.66 | 11 | 1631 | 15.14 | 0.25 |
| British Columbia | 0 | 66779 | 470.71 | 0 | 0 | 1189 | 8.71 | 0 |
| Manitoba | 166 | 29446 | 138.57 | 12.04 | 2 | 825 | 4 | 0.15 |
| New Brunswick | 12 | 1230 | 18 | 1.54 | 1 | 18 | 0.71 | 0.13 |
| Newfoundland and Labrador | 0 | 408 | 1.43 | 0 | 0 | 4 | 0 | 0 |
| Northwest Territories | 0 | 35 | 0.57 | 0 | 0 | 0 | 0 | 0 |
| Nova Scotia | 3 | 1580 | 1.43 | 0.31 | 0 | 65 | 0 | 0 |
| Nunavut | 1 | 284 | 2.43 | 2.54 | 0 | 1 | 0 | 0 |
| Ontario | 2063 | 266363 | 1968.29 | 14 | 73 | 6145 | 56 | 0.50 |
| Prince Edward Island | 0 | 111 | 0.14 | 0 | 0 | 0 | 0 | 0 |
| Quebec | 1367 | 261360 | 1312 | 15.94 | 46 | 9763 | 46.57 | 0.54 |
| Saskatchewan | 588 | 23626 | 244.14 | 49.89 | 15 | 300 | 7.14 | 1.27 |
| Yukon | 0 | 70 | 0 | 0 | 0 | 1 | 0 | 0 |
| **Canada** | 4583 | 775031 | 3952.71 | 12.06 | 148 | 19942 | 138.29 | 0.39 |

**Table S5. Static surveillance metrics for the week of 1/31/2021-2/6/2021**

| Province | New cases | Cumulative cases | 7-day moving average | Infection rate | Deaths | Cumulative deaths | 7-day moving average of death | Death rate |
| --- | --- | --- | --- | --- | --- | --- | --- | --- |
| Alberta | 348 | 126408 | 381.29 | 7.87 | 12 | 1705 | 10.57 | 0.27 |
| British Columbia | 0 | 69716 | 419.57 | 0 | 0 | 1246 | 8.14 | 0 |
| Manitoba | 80 | 30158 | 101.71 | 5.80 | 4 | 842 | 2.43 | 0.29 |
| New Brunswick | 12 | 1337 | 15.29 | 1.54 | 2 | 20 | 0.29 | 0.26 |
| Newfoundland and Labrador | 3 | 415 | 1 | 0.57 | 0 | 4 | 0 | 0 |
| Northwest Territories | 0 | 36 | 0.14 | 0 | 0 | 0 | 0 | 0 |
| Nova Scotia | 0 | 1584 | 0.57 | 0 | 0 | 65 | 0 | 0 |
| Nunavut | 3 | 299 | 2.14 | 7.62 | 0 | 1 | 0 | 0 |
| Ontario | 1388 | 276718 | 1479.29 | 9.42 | 45 | 6483 | 48.29 | 0.31 |
| Prince Edward Island | 0 | 112 | 0.14 | 0 | 0 | 0 | 0 | 0 |
| Quebec | 1204 | 268977 | 1088.14 | 14.04 | 26 | 9999 | 33.71 | 0.30 |
| Saskatchewan | 263 | 25209 | 226.14 | 22.31 | 4 | 336 | 5.14 | 0.34 |
| Yukon | 0 | 70 | 0 | 0 | 0 | 1 | 0 | 0 |
| **Canada** | 3301 | 801039 | 3154.86 | 8.69 | 93 | 20702 | 108.57 | 0.24 |

**Table S6. Novel surveillance metrics for the week of 1/3/2021-1/9/2021**

| Province | Speed: daily positives per 100,000 (weekly average of new daily cases per 100,000) | Acceleration: day-to-day change in the number of positives per day, weekly average, per 100,000 | Jerk: week-over-week change in acceleration, per 100,000 | 7-day persistence effect on speed (number of new cases per day per 100,000 attributed to new cases 7 days ago) |
| --- | --- | --- | --- | --- |
| Alberta | 22.01 | 0.29 | 0.67 | 10.76 |
| British Columbia | 12.88 | 0 | -1.71 | 3.11 |
| Manitoba | 11.81 | -1.27 | -3.56 | 4.81 |
| New Brunswick | 2.82 | 0.37 | 0.07 | 0.13 |
| Newfoundland and Labrador | 0.05 | 0 | 0 | 0.05 |
| Northwest Territories | 0 | 0 | 0 | 0 |
| Nova Scotia | 0.44 | -0.15 | -0.18 | 0.16 |
| Nunavut | 0 | 0 | 0 | 0.12 |
| Ontario | 23.11 | -2.32 | -6.44 | 5.97 |
| Prince Edward Island | 0.54 | 0 | -0.45 | 0.06 |
| Quebec | 39.31 | 5.21 | 0.90 | 9.26 |
| Saskatchewan | 23.73 | -2.01 | -6.08 | 6.39 |
| Yukon | 3.40 | 0 | -0.34 | 0 |
| **Canada** | 23.37 | 0.20 | -2.77 | 6.46 |

**Table S7. Novel surveillance metrics for the week of of 1/10/2021-1/16/2021**

| Province | Speed: daily positives per 100,000 (weekly average of new daily cases per 100,000) | Acceleration: day-to-day change in the number of positives per day, weekly average, per 100,000 | Jerk: week-over-week change in acceleration, per 100,000 | 7-day persistence effect on speed (number of new cases per day per 100,000 attributed to new cases 7 days ago) |
| --- | --- | --- | --- | --- |
| Alberta | 17.59 | -0.88 | 0.41 | 7.30 |
| British Columbia | 9.67 | 0 | 0.30 | 4.27 |
| Manitoba | 11.97 | -0.27 | 0.04 | 3.91 |
| New Brunswick | 2.67 | -0.05 | -0.18 | 0.93 |
| Newfoundland and Labrador | 0.08 | 0 | -0.03 | 0.02 |
| Northwest Territories | 0.32 | 0 | -0.32 | 0 |
| Nova Scotia | 0.36 | 0.01 | 0.01 | 0.15 |
| Nunavut | 0 | 0 | 0 | 0 |
| Ontario | 21.84 | -0.38 | 0.84 | 7.66 |
| Prince Edward Island | 0.18 | 0 | 0.45 | 0.18 |
| Quebec | 24.55 | -1.50 | -0.39 | 13.03 |
| Saskatchewan | 26.45 | -0.72 | -1.32 | 7.87 |
| Yukon | 0 | 0 | 0.34 | 1.13 |
| **Canada** | 18.69 | -0.62 | 0.28 | 7.75 |

**Table S8. Novel surveillance metrics for the week of 1/17/2021-1/23/2021**

| Province | Speed: daily positives per 100,000 (weekly average of new daily cases per 100,000) | Acceleration: day-to-day change in the number of positives per day, weekly average, per 100,000 | Jerk: week-over-week change in acceleration, per 100,000 | 7-day persistence effect on speed (number of new cases per day per 100,000 attributed to new cases 7 days ago) |
| --- | --- | --- | --- | --- |
| Alberta | 13.71 | -0.47 | -0.01 | 5.83 |
| British Columbia | 9.34 | 0 | 0 | 3.21 |
| Manitoba | 11.95 | 0.40 | 0.61 | 3.97 |
| New Brunswick | 3.53 | -0.18 | -0.27 | 0.88 |
| Newfoundland and Labrador | 0.08 | 0 | 0 | 0.03 |
| Northwest Territories | 1.90 | 0 | 0.32 | 0.10 |
| Nova Scotia | 0.23 | -0.06 | -0.10 | 0.12 |
| Nunavut | 0.36 | 0 | -0.36 | 0 |
| Ontario | 17.67 | -0.68 | -0.35 | 7.24 |
| Prince Edward Island | 0.54 | 0 | 0 | 0.06 |
| Quebec | 18.67 | -0.90 | -0.42 | 8.14 |
| Saskatchewan | 23.42 | 0.05 | 1.03 | 8.77 |
| Yukon | 0 | 0 | 0 | 0 |
| **Canada** | 15.17 | -0.51 | -0.19 | 6.19 |

**Table S9. Novel surveillance metrics for the week of 1/24/2021-1/30/2021**

| Province | Speed: daily positives per 100,000 (weekly average of new daily cases per 100,000) | Acceleration: day-to-day change in the number of positives per day, weekly average, per 100,000 | Jerk: week-over-week change in acceleration, per 100,000 | 7-day persistence effect on speed (number of new cases per day per 100,000 attributed to new cases 7 days ago) |
| --- | --- | --- | --- | --- |
| Alberta | 11.04 | -0.61 | -0.29 | 4.54 |
| British Columbia | 9.14 | 0 | -0.02 | 3.10 |
| Manitoba | 10.05 | -0.52 | -0.32 | 3.96 |
| New Brunswick | 2.30 | -0.09 | 0.16 | 1.17 |
| Newfoundland and Labrador | 0.27 | 0 | -0.08 | 0.03 |
| Northwest Territories | 1.27 | 0 | -1.27 | 0.63 |
| Nova Scotia | 0.15 | 0.04 | 0.10 | 0.08 |
| Nunavut | 6.17 | 0.36 | 0.73 | 0.12 |
| Ontario | 13.36 | -0.29 | 0.51 | 5.86 |
| Prince Edward Island | 0.09 | 0 | -0.09 | 0.18 |
| Quebec | 15.30 | -0.53 | 0.03 | 6.19 |
| Saskatchewan | 20.71 | 3.81 | 2.75 | 7.76 |
| Yukon | 0 | 0 | 0 | 0 |
| **Canada** | 12.22 | -0.20 | 0.25 | 5.03 |

**Table S10. Novel surveillance metrics for the week of 1/31/2021-2/6/2021**

| Province | Speed: daily positives per 100,000 (weekly average of new daily cases per 100,000) | Acceleration: day-to-day change in the number of positives per day, weekly average, per 100,000 | Jerk: week-over-week change in acceleration, per 100,000 | 7-day persistence effect on speed (number of new cases per day per 100,000 attributed to new cases 7 days ago) |
| --- | --- | --- | --- | --- |
| Alberta | 8.62 | -0.11 | 0.36 | 4.01 |
| British Columbia | 8.15 | 0 | 0.12 | 3.20 |
| Manitoba | 7.37 | -0.89 | -0.46 | 3.71 |
| New Brunswick | 1.96 | 0 | 0.16 | 0.82 |
| Newfoundland and Labrador | 0.19 | 0.08 | 0.16 | 0.10 |
| Northwest Territories | 0.32 | 0 | 1.27 | 0.57 |
| Nova Scotia | 0.06 | -0.04 | -0.03 | 0.06 |
| Nunavut | 5.45 | 0.73 | 0.73 | 2.09 |
| Ontario | 10.04 | -0.65 | -0.49 | 4.87 |
| Prince Edward Island | 0.09 | 0 | 0.09 | 0.04 |
| Quebec | 12.69 | -0.27 | 0.05 | 5.59 |
| Saskatchewan | 19.19 | -3.94 | -2.41 | 8.24 |
| Yukon | 0 | 0 | 0 | 0 |
| **Canada** | 9.78 | -0.48 | -0.20 | 4.46 |
